# Young versus aged microbiota transplants to germ-free mice: increased short-chain fatty acids and improved cognitive performance

**DOI:** 10.1080/19490976.2020.1814107

**Published:** 2020-09-08

**Authors:** Juneyoung Lee, Venugopal R. Venna, David J. Durgan, Huanan Shi, Jacob Hudobenko, Nagireddy Putluri, Joseph Petrosino, Louise D. McCullough, Robert M. Bryan

**Affiliations:** a Department of Neurology McGovern Medical School, University of Texas Health Science Center at Houston, Houston, TX, USA; b Departments of Anesthesiology, Baylor College of Medicine, Houston, TX, USA; c Molecular Physiology and Biophysics, Baylor College of Medicine, Houston, TX, USA; d Molecular and Cellular Biology, Baylor College of Medicine, Houston, TX, USA; e Molecular Virology and Microbiology, Baylor College of Medicine, Houston, TX, US

**Keywords:** Aging, gut microbiome, short-chain fatty acids, acetate, propionate, butyrate, inflammation, germ-free mice, cognitive decline

## Abstract

Aging is associated with cognitive decline and decreased concentrations of short-chain fatty acids (SCFAs) in the gut. SCFAs are significant in that they are protective to the gut and other organs. We tested the hypothesis that the aged gut microbiome alone is sufficient to decrease SCFAs in the host and produce cognitive decline. Fecal transplant gavages (FTGs) from aged (18–20 months) or young (2–3 months) male C57BL/6 mice into germ-free male C57BL/6 mice (N = 11 per group) were initiated at ~3 months of age. Fecal samples were collected and behavioral testing was performed over the study period. Bacterial community structures and relative abundances were measured in fecal samples by sequencing the bacterial 16S ribosomal RNA gene. Mice with aged and young microbiomes showed clear differences in bacterial β diversity at 30, 60, and 90 d (*P* = .001 for each) after FTGs. The fecal SCFAs, acetate, propionate, and butyrate (microbiome effect, *P* < .01 for each) were decreased in mice with an aged microbiome. Mice with an aged microbiome demonstrated depressive-like behavior, impaired short-term memory, and impaired spatial memory over the 3 months following the initial FTG as assessed by the tail suspension (*P* = .008), the novel object recognition (*P* < .001), and the Barnes Maze (*P* = .030) tests, respectively. We conclude that an aged microbiome alone is sufficient to decrease SCFAs in the host and to produce cognitive decline.

## Introduction

In the past decade, it has become apparent that the composition, diversity, and function of the gut microbiome changes in later life, resulting in chronic immune dysregulation and enhanced susceptibility to age-associated diseases.^1–7^ One of the many changes associated with aging is a decrease in short-chain fatty acids (SCFAs) in the gut.^5,8-10^ This is significant since the predominant source of SCFAs in the host are derived from bacterial fermentation of non-digestible carbohydrates.^11,12^ Furthermore, SCFAs, primarily acetate, propionate, and butyrate are considered to be highly beneficial or protective to the host by either activating G protein-coupled receptors or by inhibiting histone deacetylases.^12–16^ Working through either or both of the pathways, SCFAs stabilize the gut epithelial barrier, increase thickness of the protective mucus layer, modulate cytokine secretion, promote regulatory T cell generation, modulate antibody secretion, and inhibit NF-kB.^12–17^ Furthermore, beneficial effects of SCFAs have been reported in organs outside and distant to the gut. For example, SCFA-producing bacteria, and SCFAs, control brain microglia function^18-20^ and are instrumental in development of the blood-brain barrier.^21^

It is not fully known if the decreases in SCFAs in the aged gut is strictly a function of the bacteria or is an interaction between the bacteria and the aged host. Furthermore, studies attempting to manipulate SCFAs in the gut through fecal transplant gavages (FTG) rely on antibiotics to significantly decrease the resident bacteria in the host.^9^ It is not known how the antibiotic affects the FTGs and, thus, the SCFA-producing bacteria and SCFAs in the gut.

Therefore, we tested the hypothesis that the aged gut microbiome alone is sufficient to decrease SCFAs compared to that of a young microbiome. In addition, we also tested the hypothesis that the aged microbiome alone is sufficient to produce cognitive decline. In order to test our hypotheses, we conducted FTGs from young and aged mice into young germ-free (GF) mice. The advantage is that recipient GF mice are all the same age, have identical physiological states, have had no prior exposure to antibiotics, and have no residual gut microbiota to compete with the microbiota used in the FTGs.

## Results

**Figure 1.**
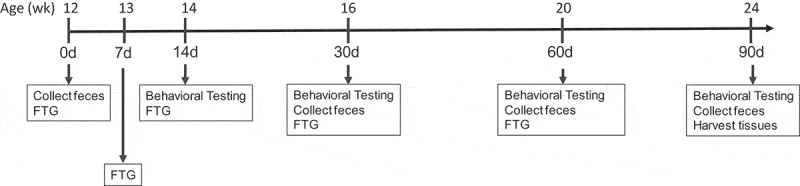
The time line for the studies in germ-free mice. Following FTG (fecal transplant gavage) mice with young and aged FTG were housed separately in a conventional animal holding room

At 12 weeks of age 11 GF mice received FTGs from young mice (2–3 months) and 11 GF mice received FTGs from aged mice (18–20 months, Figure 1). At 24 weeks of age (90 d post-FTG), when the study was terminated, the previously GF mice with young and aged microbiomes had gained 3.3 ± 0.4 g and 2.9 ± 0.4 g, respectively (not significant; data not shown). Fecal samples were collected at 30, 60, and 90 d after the initial FTG for 16S ribosomal RNA (rRNA) gene analysis in order to compare microbiomes.

### Beta diversity in mice with aged and young microbiomes

**Figure 2.**
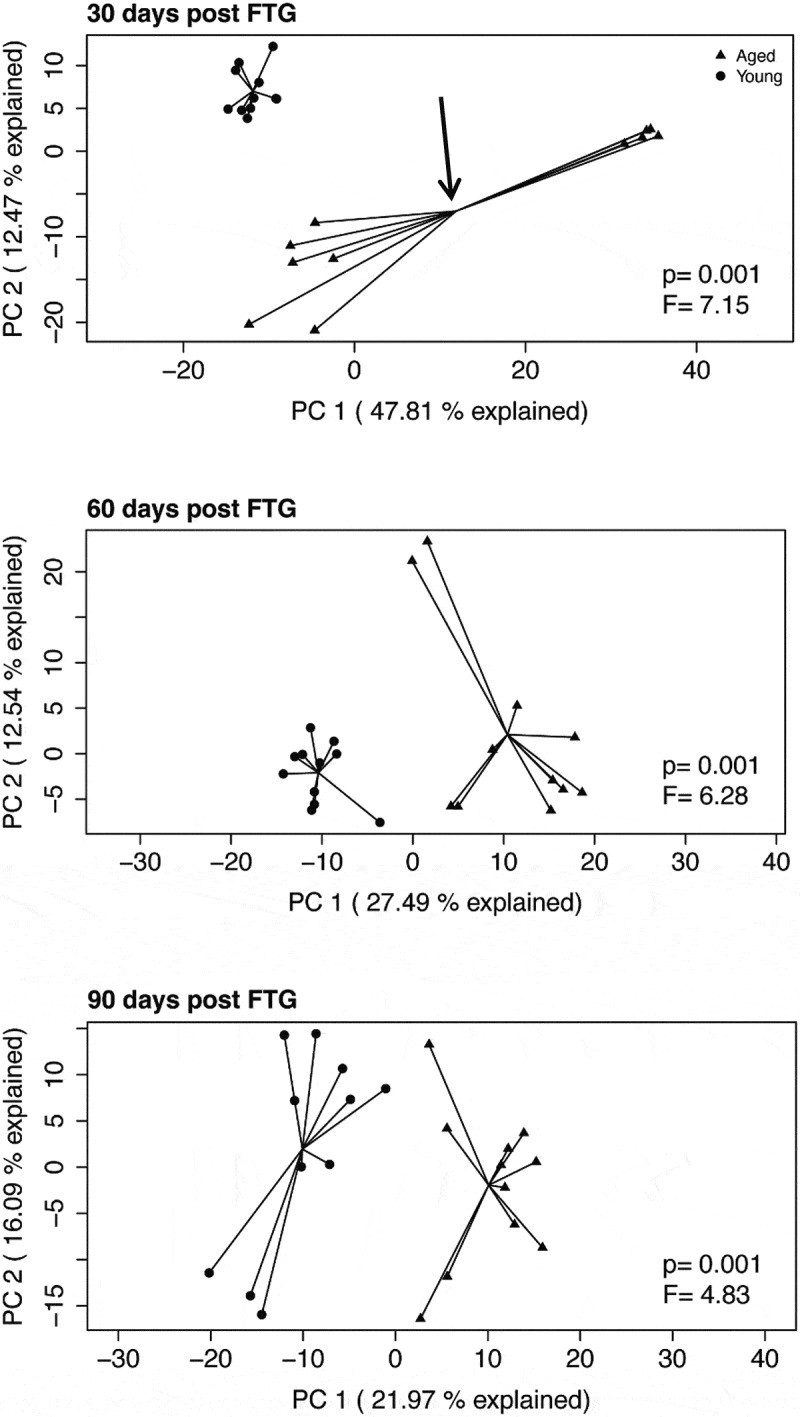
Principal component analyses (PCAs) of the Aitchison distance, a measure of beta diversity, in mice with young and aged microbiomes at 30, 60, and 90 d after the initial fecal transplant gavage. PERMANOVA F statistics and *P* value is within each plot. The aged group at 30 d (top panel, depicted by arrow pointing to the group centroid) has data points that cluster into two groups. This clustering is discussed in the manuscript

Figure 2 shows principal component analyses (PCAs) of the Aitchison distance, a measure of beta diversity, in mice with young or aged microbiomes at 30, 60, and 90 d post FTG. Note the clear separation between the young and aged microbiomes at each of the three time points (PERMANOVA, *P* = .001 for each time). Also, note the separation in the points on the plot for the aged microbiome at 30 d; 6 samples cluster to the left of the centroid and 5 samples cluster to the right of the centroid (Figure 2, arrow in top panel). The separation in the aged group at 30 d was traced back to a single cage (points to the right of the centroid). In the group housed in a single cage, there was an expansion of *Parabacteroides* and possibly *Akkermansia* (see bracket in Supplemental Figure II).

### Alpha diversity with aged and young microbiomes

Alpha diversities for fecal samples were calculated using Shannon, Simpson, and Chao1 indices in mice with aged and young microbiomes (Supplemental Figure I). All three indices were significantly decreased in mice with an aged microbiome at 30, 60, and 90 d after the initial FTG. These indices suggest that there is both a decrease in diversity and possibly a decrease in evenness with an aged microbiome.

### Bacterial abundance with aged and young microbiomes

**Figure 3.**
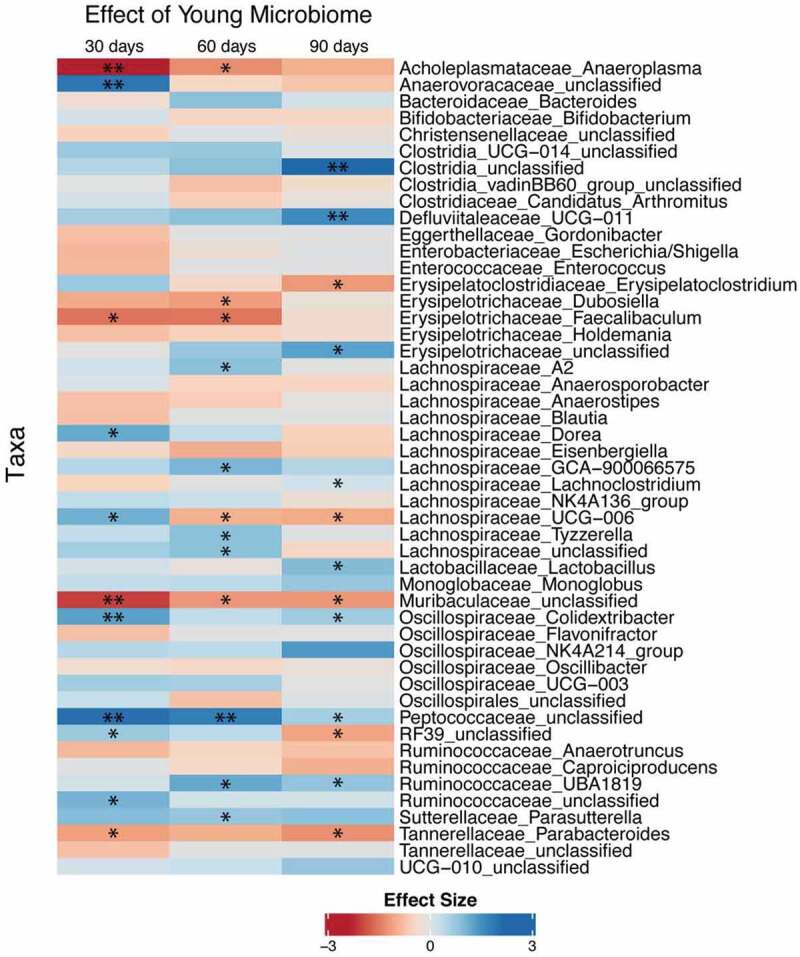
Differential abundance of taxa from GF mice after fecal transplant gavage with an aged or young gut microbiome. Only taxa showing *P* < .2 in at least one of the time points were displayed. Statistical analysis was conducted after a centered log-ratio transformation using Kruskal–Wallis test with post hoc Benjamini-Hochberg correction. N = 11 per group. * and ** *P* < .05 and 0.01 respectively

Figure 3 shows differences in the abundance of bacterial taxa in feces resulting from a young FTG at 30, 60, and 90 d compared to that in mice with an aged FTG. An unclassified genus or genera in the *Muribaculaceae* family significantly decrease at all three time points and an unclassified genus or genera in the *Peptococcaceae* family significantly increased at all three time points in mice with a young microbiome compared to that with an aged microbiome. In some instances, the young microbiome was either consistently increased or consistently decreased across the time points, although statistical significance was not achieved for all of the times. *Lachnospiraceae_UCG-006* significantly decreased at 60 and 90 d. Mice with young microbiomes showed taxa increases in SCFA or putative SCFA producers including genera in the family, *Lachnospiraceae*, an unclassified genus or genera in the *Peptococcaceae* family, *Ruminococcaceae_UBA1819*, and *Oscillospiraceae_Colidextribacter*.^22–24^ Although some bacteria capable of producing SCFAs increased in mice with an aged microbiome, the overall effect suggests more abundant SCFA-producing taxa in mice with a young microbiome. A stacked bar chart of taxa abundance for individual mice is shown in Supplemental Figure II.

**Figure 4.**
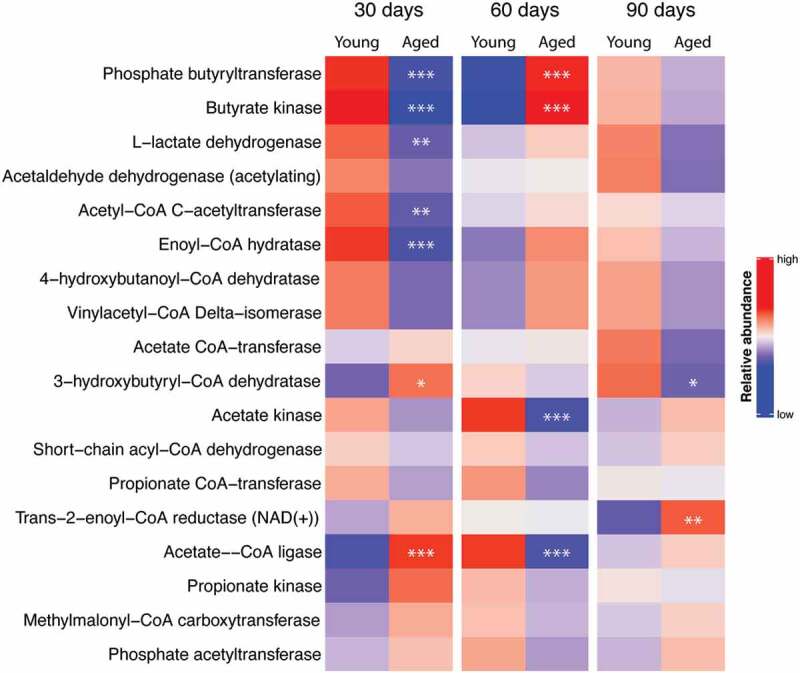
Heat map of relative abundance for bacterial enzymes used in the metabolism of acetate, propionate, and butyrate. Predictions were made from feces of mice collected 30, 60, and 90 d after the initial fecal transp gavage using data from 16S ribosomal RNA gene analysis and the software program, PICRUSt2. *, **, and *** *P* < .05, <0.01, and <0.001 respectively, N = 11 per group)

Functional abundance of important bacterial enzymes used in the metabolism of acetate, propionate, and butyrate was determined from 16S rRNA gene analysis of feces harvested at 30, 60, and 90 d using the software program, PICRUSt2 Figure 4. This heat map does not necessarily predict if SCFAs should be decreased in mice with an aged microbiome since many of these enzymatic reactions can operate in either the forward or reverse direction. However, the heat map does indicate that enzymes involved in SCFAs metabolism in gut are different in the 2 groups of mice and predicts potential differences of the SCFA production with young and aged microbiomes. Using PICRUSt2 we were able to determine which of the bacteria taxa that we found significantly different in Figure 3 contributed to the significant changes in SCFA-related enzymes shown in Figure 4.^24^ Table 1 shows the enzymes that significantly changed (column 1), the sampling day when a difference in the enzyme expression occurred (columns 2–4), the taxa potentially responsible for the change (column 5), and the effect of the taxa on increasing or decreasing the enzyme in mice with a young microbiome (column 5). Note that *Ruminococcaceae*_*UBA1819*, genera of the *Lachnospiraceae* family, an unclassified genus or genera of the *Peptococcacaeae* family, the genus, *Colidextribacter*, and an unidentified members of the class, *Bacilli*, increased at relevant times and could be responsible for the changes in enzymes involved with SCFA metabolism. The analysis of bacterial taxa in young and aged mice would predict that the microbiota in young mice secrete more SCFAs than that of mice with an aged microbiome.

**Table 1.** Table of enzyme expression showing significant differences between mice with young and aged microbiomes (column 1), day after FTG (fecal transplan gavage) when the significant change in enzyme expression occurred (columns 2, 3, and 4), and bacteria genera that significantly changed for the given day indicated in columns 2–4 with the direction of change in abundance occurring in mice with a young microbiome (column 5)

DAY AFTER FTG				
Enzyme	30	60	90	Taxa increase (↑) or decrease (↓) with young microbiome
3-HYDROXYBUTYRYL-COA DEHYDRATASE			90	*Ruminococcaceae_UBA1819 ↑*
ACETATE KINASE		60		*Lachnospiraceae_Tyzzerella↑*
		60		*Lachnospiraceae;GCA-900066575 ↑*
		60		*Lachnospiraceae_A2 ↑*
		60		*Lachnospiraceae_unclassified ↑*
		60		*Peptococcacaeae_unclassified ↑*
		60		*Ruminococcaceae_UBA1819 ↑*
		60		*Erysipelotrichaceae Dubosiella ↓*
		60		*Anaeroplasma ↓*
		60		*Lachnospiraceae_UCG-006 ↓*
		60		*Muribaculaceae_unclassified ↓*
ACETATE–COA LIGASE	30			*Bacilli_RF39_unclassified ↑*
	30	60		*Peptococcacaeae_unclassified ↑↑*
	30			*Muribaculaceae_unclassified ↓*
	30			*Parabacteroides ↓*
ACETYL-COA C-ACETYLTRANSFERASE	30			*Anaerovoraceae_unclassified ↑*
	30			*Bacilli_RF39_unclassified ↑*
	30			*Colidextribacter ↑*
	30			*Lachnospiraceae_UCG-006 ↑*
	30			*Peptococcacaeae_unclassified ↑*
	30			*Anaeroplasma ↓*
BUTYRATE KINASE	30			*Colidextribacter ↑*
		60		*Lachnospiraceae_unclassified ↑*
		60		*Lachnospiraceae_UCG-006 ↓*
ENOYL-COA HYDRATASE	30			*Anaerovoraceae_unclassified ↑*
	30			*Colidextribacter ↑*
	30			*Peptococcacaeae_unclassified ↑*
L-LACTATE DEHYDROGENASE	30			*Bacilli_RF39_unclassified ↑*
	30			*Colidextribacter ↑*
	30			*Lachnospiraceae_Dorea ↑*
	30			*Lachnospiraceae_UCG-006 ↑*
	30			*Peptococcacaeae_unclassified ↑*
	30			*Anaeroplasma ↓*

### SCFAs with aged and young microbiomes

**Figure 5.**
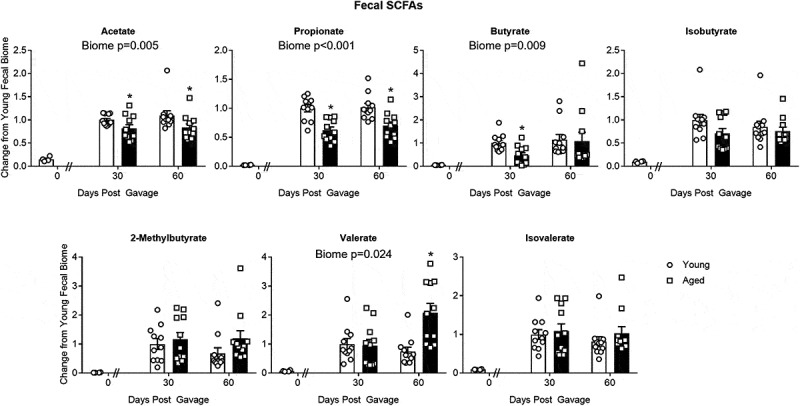
Changes in SCFA concentrations in fecal samples of mice with aged and young gut microbiomes. The “0” time point represents concentrations in the GF mice before fecal transp gavage. Note the “0” time point was not used in the statistical analysis. Data for individual SCFAs were normalized to the value obtained in the “30 day” group having a young microbiome. Significant main effects of the microbiome (aged or young) when present are shown in each graph. * *P* < .05 compared to the young microbiome of the same time point (Holm-Sidak post hoc test, N = 11 per group). See Supplemental Table I for more statistical details. Note that the SCFAs in the 30 d aged group showed a dichotomy as described under beta diversity above. Dichotomy in the SCFAs did not occur in any other group

Figure 5 shows relative SCFA concentration in feces at 0, 30 and 60 d after the initial gavage. Note that concentrations of SCFAs were substantially lower in the GF mice at baseline “0 days” as would be expected in animals lacking gut bacteria. Compared to mice with a young microbiome, mice with an aged microbiome showed significant decreases in fecal acetate (*P* = .005), propionate (*P* < .001), and butyrate concentrations (*P* = .009; N = 11 per group; two-way repeated measures ANOVA). [Note: Supplemental Table I provides a more complete analysis for all two-way ANOVA in this study]. Holm-Sidak post hoc analysis was used to compare individual groups where “*” represents a significant difference in mice with an aged microbiome compared to a young microbiome at the same time point. With the exception of acetate and possibly butyrate, cecal concentrations of SCFAs at 90 d were similarly decreased by ~50% (Supplemental Figure III). Note that the SCFAs in the 30 d aged group showed a dichotomy as described under beta diversity above. The five samples that are to the right of the centroid in Figure 2 show significant decreases for all SCFAs compared to the other six samples, which are left of the centroid. Dichotomy in the SCFAs did not occur in any other group.

### Behavioral testing with aged and young microbiomes

**Figure 6.**
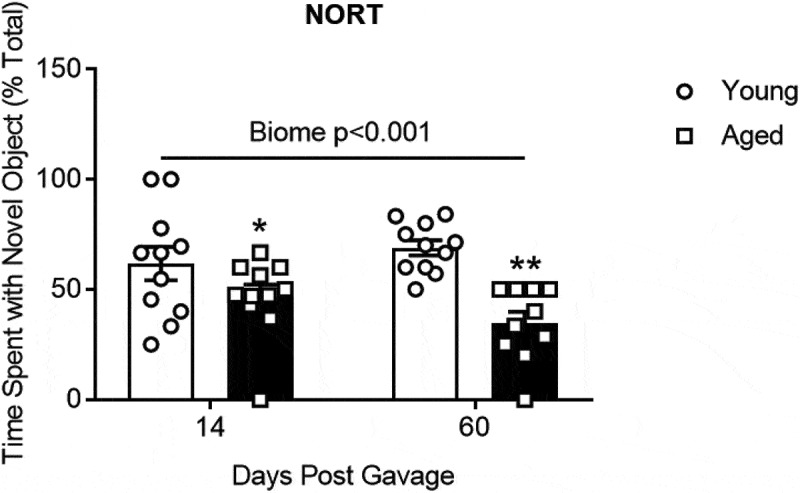
The effects of aged or young gut microbiome on short-term memory recall using the novel object recognition test (NORT). Duration of time spent at the novel object as a percent of the total time. Significant main effect of gut microbiome using 2 way repeated measures ANOVA *P* < .001, N = 10–11. *, ** *P* < .05 and <0.001 compared to young biome of the same time (post hoc Holm-Sidak test). See Supplemental Table I for more statistical details. Note: one mouse with an aged microbiome failed to explore at 60 d and was omitted from the analysis

Figure 6 summarizes the results of the novel object recognition test which is a measure of short-term memory. With short-term recall mice will spend more time at a novel object than a familiar object. Results of the novel object recognition test demonstrate that mice with a young microbiome spend more time on the novel object than mice with aged microbiomes (*P* < .001; N = 10–11 per group; two-way repeated measures ANOVA, see Supplemental Table I).

**Figure 7.**
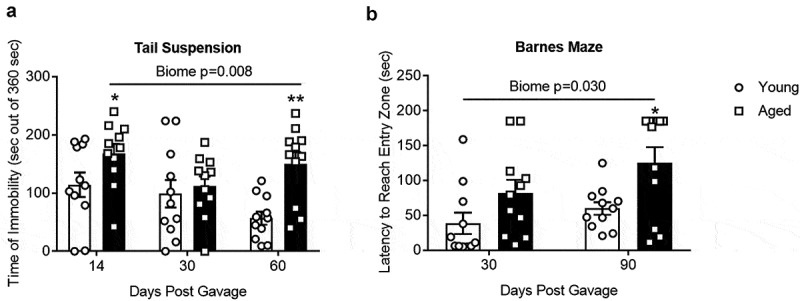
(a) Tail suspension test and (b) Barnes Maze. There was a significant main effect of the gut microbiome (*P* = .008 and 0.030 for A and B respectively, (N = 9–11). *,** *P* < .05 and 0.001 compared to young biome at the same time point (Holm-Sidak post hoc analysis). See Supplemental Table I for more statistical details. Note that the time of immobility in the 30 d aged group showed a dichotomy as described in the text. None of the other behavioral tests regardless of time after the initial gavage showed a dichotomy

Figure 7a shows the results of the tail suspension test, a measure of depressive-like behavior. Mice that spend less time attempting to escape (more immobility time) are considered to have a more depressive-like phenotype. Mice with an aged microbiome had more immobility time (*P* = .008; N = 9–11; two-way repeated measures ANOVA, see Supplemental Table I) compared to those with a young microbiome. Post hoc Holm-Sidak analysis also demonstrated significant effects of the aged microbiome (“*” and “**” represent *P* < .05 and 0.001, respectively) compared to the group with the young microbiome for the same time period. Note that the time of immobility in the 30 d aged group showed a dichotomy as described under beta diversity above. The five samples to the right of the centroid in Figure 2have statistically significant increases in time of immobility compared to the other six samples, which are left of the centroid (149 ± 10 versus 83 ± 21, *P* = .027). None of the other behavioral tests regardless of time after the initial gavage showed a dichotomy based on mice housing. Figure 7b shows the results of the Barnes Maze test, a test for spatial memory. Overall mice with an aged microbiome showed a longer latency to reach the entry zone for an escape hole (*P* = .030; N = 11; two-way repeated measures ANOVA).

Finally, there were no differences in distance traveled in an open field test or the time mice could hang on a wire without falling (Supplemental Figure IV, A and B), indicating that neither activity nor strength should be factors affecting performance in other tests in the aged mice. In the open field test, there were no differences in either the total moving time or the velocity of movement between mice with young and aged microbiomes at any of times following the days after the initial FTG.

### Inflammatory markers in brain and plasma

Flow cytometry was conducted using brains of mice with a young or aged microbiome. Supplemental figure V shows the gating strategy and corresponding results for flow cytometry studies from brains 90 d after the initial FTG. There were no statistical differences in the number of CD45^+^ cells (leukocyte), CD4^+^ helper T cells, CD8^+^ cytotoxic T cells, and γδ T cells (CD45^+^CD4^−^CD8^−^TCR γδ^+^) in brains from mice with a young and aged microbiome (N = 5 per group). Supplemental Figure VI shows plasma inflammatory cytokines in the plasma of mice 90 d after the initial FTG (N = 6–10 for each group). There were no significant differences in IL-5, IL-6, IL-9, IL-10, IL-12 (p40), eotaxin, granulocyte colony-stimulating factor (G-CSF), granulocyte-macrophage colony-stimulating factor (GM-CSF), IFN-γ, keratinocyte chemoattractant (KC, also known as CXCL1) or, monocyte chemoattractant protein-1 (MCP-1), macrophage inflammatory protein-1α (MIP-1α), RANTES, or TNF-α. We note that inflammatory markers in brain and plasma (Supplemental Figures V and VI) were only measured at the termination of the study, 90 d after the initial FTG. Thus, we do not know if the inflammatory status was different between groups at earlier time points.

## Discussion

The gut harbors a diverse and complex community of microbiota that changes in composition and diversity with the aging process.^5,8,25^ This age-related change can have detrimental effects on the host by interfering with immune and physiological functions in the gastrointestinal system as well as tissue and organs distant to the gut.^26,27^ The underlying pathological basis of the aged microbiome appears to involve a shifting environment that is more conducive for the growth of detrimental bacteria and less conducive for the growth of beneficial bacteria. One result of this shift in the gut environment involves a decrease in SCFAs, fatty acids less than 6 carbons that serve as important signaling molecules and energy substrate in the host.^5,8-10^ Interestingly, the SCFAs are almost exclusively derived from bacterial fermentation of non-digestible carbohydrates.^11,12^

It is not fully known if the decrease in SCFAs in the aged gut is strictly a function of the bacteria, is strongly influenced by prior antibiotic treatment as used in FTG studies, or is a combination of the bacterial interaction with the aged host. From the present study, we demonstrate that (1) the aged gut microbiome alone is sufficient to produce decreases in SCFAs in GF mice; and (2) the aged microbiome alone is sufficient to produce cognitive decline.

We report a dichotomy in results for beta diversity in the mice with an aged microbiome at 30 d. The dichotomy could be traced back to mice housed in a single cage. This cage also showed decreased SCFAs and significantly longer immobilization times for the tail suspension compared to the mice housed in other cages (points left of the centroid). This dichotomy occurred only at 30 d in mice with an aged microbiome; it did not occur at other time points or in any other group. We have no explanation for this dichotomy. None of the behavioral tests conducted at 14 d, including the tail suspension test which did show separation at 30 d, showed a dichotomy. After 30 d, dichotomy was not seen in the microbiome, SCFAs, or in any behavioral test. Perhaps the FTG immediately after feces collection and behavioral testing at 30 d stabilized the gut microbiome in this group.

(1) The aged gut microbiome alone is sufficient to produce decreases in SCFAs in GF mice. In GF mice, SCFA concentrations were much lower than after FTG from either young or aged mice (Figure 5, compare “0” time point to other times) as would be expected in animals lacking gut bacteria. After FTG, SCFAs in feces and cecal content are generally decreased in mice with an aged microbiome compared to mice with a young microbiome (Figure 5 and Supplemental Figure III). Overall, these findings highlight the importance of gut bacteria in SCFA production and imply many of the pathological changes with aging might be attributed to the gut and the gut microbiome. Data from analysis of bacteria taxa, functional expression of bacterial enzymes related to SCFA production, and SCFA concentrations in feces and cecal content act to confirm and amplify the relationship between the gut microbiota and SCFAs in the gut.

Consistent with the changes in SCFAs in feces and cecal content, SCFA-producing bacteria were more abundant in GF mice with a young microbiome compared to mice with an aged microbiome Figure 3. For example, mice with young microbiomes showed taxa increases in SCFA or putative SCFA producers including genera in the family, *Lachnospiraceae*, an unclassified genus or genera in the *Peptococcaceae* family, *Ruminococcaceae_UBA1819*, and *Oscillospiraceae_Colidextribacter*.^22–24,28-30^ In general, our results are in agreement with a study where aged and young donor microbiomes were transplanted into sexually mature male mice after antibiotic treatment.^31^

Our results are consistent with the preponderance of previous studies reporting decreases in SCFAs with aging^2,9,10,12^ but at odds with a recent study reporting that aged microbiomes transplanted into GF mice either increased fecal butyrate or had no significant effect on fecal butyrate depending on the age when GF mice where inoculated.^32^ Of note, SCFAs other than butyrate were not measured in this latter study. This study also reported that the aged microbiome increased neurogenesis in the hippocampus and enhanced growth in the intestines; changes which are not normally associated with aging. We have no explanation for the apparent discrepancies between the results of our study and those by Kundu et al.^32^ Given the importance of the subject, it is imperative that future studies address these inconsistencies to help explain the significance of the “aged” microbiome.

In a related study, Boehme et al.^26^ reported that a prebiotic supplementation (oligofructose-enriched inulin) dampened a heightened inflammatory responses in middle-aged mice (10 months). Interestingly, the study implicated prebiotic changes in cecal SCFA as a potential mechanism for altering the cognitive and immune responses. Although the changes we found in SCFA with aging mice cannot necessarily be extrapolated from the studies in middle aged mice,^26^ there are parallels that support an important role for SCFAs in improving cognitive function.

(2) The aged microbiome alone is sufficient to produce cognitive decline. The aging process increases the probability for cognitive decline in humans and animals.^9,25,33,34^ Consistent with this idea, GF mice receiving an aged gut microbiome showed significantly poorer performance on tests involving memory recall (novel object recognition test, Figure 6) or spatial memory (Barnes Maze, Figure 7b). Furthermore, mice with aged microbiomes showed more depressive-like behavior as assessed by the tail suspension test Figure 7a. We note that there were no differences in distance traveled in an open field test and the time mice could hang to a wire without falling (Supplemental Figure IV, A and B) indicating that activity and/or strength were not factors affecting performance in other tests in the aged mice.

Our study does have a number of limitations: first, while the data support our hypothesis as stated, we recognize that the correlation between SCFAs and cognitive performance does not prove causality. Further studies will be needed to understand the full relationship, if any, for a role of SCFAs in improving cognitive performance in aging. Second, we did not analyze the microglia in our flow cytometry analysis. The state of the microglia could have potentially provided valuable insight into the neurological effects of aged and young microbiome. Third, we pooled fecal samples from young mice and pooled samples from aged mice to generate suspensions for gavaging into the GF mice. We recognize that an outlier microbiome could skew the composition of the pooled sample or create a microbiome composition that does not naturally occur. Fourth, while the use of GF mice does have advantages, we do point out that GF mice have inherent differences compared to conventionally raised mice. That is, many of the physiological processes are considered abnormal prior to FTG. Therefore, the responses to the FTG have the potential to be influenced by the altered physiological state.

In summary, our study shows that transferring an aged microbiome into GF mice recapitulates the phenotypic changes that occur with aging. That is, cognitive performance is impaired, the gut microbiome is altered, and SCFA production is significantly decreased. Analysis of bacterial abundance of SCFA-producing bacteria in feces and functional abundance of important enzymes regulating production and/or degradation of SCFAs confirms this observation. We conclude that the nature of the microbiome and not an interaction between the gut microbiome and the aged host or prior treatment with antibiotics is, at least partially, responsible for the decrease in SCFA-producing bacteria and fecal and cecal SCFA concentrations. Furthermore, the aged microbiome alone is sufficient to produce cognitive decline.

## Materials and methods

### Animals

This study was performed under the guidelines of the National Institute of Health and all experiments were approved by the Institutional Animal Care and Use Committees at the University of Texas Health Science Center at Houston (UTHSCH) and the Baylor College of Medicine. Given the differences in the microbiome and inflammatory responses between male and females, sex must be treated in separate groups. We included only males in the present study since inclusion of both sexes would be prohibitive for a single study. C57BL/6-GF male mice (12 weeks) were obtained from the Baylor College of Medicine Gnotobiotic Rodent Facility and shipped in autoclaved shipping crates to UTHSCH. The crates were assembled, bedded with Alpha-Dri bedding and autoclaved with the lid closed. The mice were removed from the isolator in the gnotobiotic facility using sterile transfer bags, housed in the shipping crates under a biosafety cabinet, and immediately shipped. The two medical schools are only a few hundred yard apart precluding lengthy and stressful travel. Upon arrival at UTHSCH, fecal samples were collected to confirm the absence of bacteria in the gut. To assess the “germ-free” status of the mice upon arrival, an internal standard was serially diluted and the copy number of the 16S rRNA gene in feces from each transfer crate was analyzed using qPCR. 16S rRNA gene was not detected in feces from any of the transfer crates confirming that the mice were germ-free. Once the aseptic crates were opened at the UTHSCH, the mice were transferred to autoclaved cages, provided with autoclave chow and water, and maintained in a conventional animal holding room.

### Fecal transplant gavage (FTG)

Fresh fecal samples were collected at 9–10 am from wild-type young (2–3 months) or aged (18–20 months) donor male mice (N = 5–10 per group). The samples were pooled and immediately homogenized using ice-cold PBS (120 mg feces/1 ml) followed by centrifugation at 800 g for 3 min at 4°C. The supernatant was used for transplantation. One hundred microliters of the fecal supernatant was gavaged on arrival and at day 7, 14, 30, and 60 Figure 1. Mice were housed with other mice receiving the same FTG and placed in cages with filtered tops. Cages with aged FTG mice were separated from the young FTG mice but were housed in the same conventional animal holding room at the UTHSCH. Precautions were taken to avoid cross contamination between groups during behavioral testing and when changing cages, food, water, and bedding.

### Experimental protocol

The time line for the study is shown in Figure 1 with each collection time noted. Bacterial 16S rRNA gene and SCFAs were analyzed from feces at 30, 60, and 90 d and from cecal content at 90 d after the initial FTG. Tissues were harvested at 90 d after the initial gavage.

### 16S rRNA sequencing

Fecal samples were collected from the recipient mice and immediately frozen at −80°C. 16S rRNA sequencing was performed on collected fecal samples. DNA was extracted using MO BIO PowerMag Soil DNA Isolation Kit (MO BIO Laboratories), according to the manufacturer’s protocol. 16S rRNA gene sequence libraries were generated using the V4 region primers 515 F and 806 R on the Illumina MiSeq platform.

*Taxonomic analysis*: The raw data files in binary base call (BCL) format created by the MiSeq run were converted into FASTQ format using the Illumina ‘bcl2fastq’ software. Raw pair-ended 16s rRNA sequences (FASTQ) were demultiplexed by QIIME2 framework core function.^35^ Reads were de-noised and merged into amplicon sequence variants (ASVs) by DADA2 pipeline in R.^36,37^ Taxonomic annotations were also generated against DADA2-formatted training FASTA files derived from SILVA138 Database.^38^ ASVs with identical taxonomic assignment were grouped into taxonomic bins. Alpha diversity (Shannon Index, Simpson Index and Chao1 Index) was calculated using R package Vegan.^39^ Student’s t-test was used to compare alpha diversity at each time point. For principal component analysis (PCA), permutational multivariate analysis of variance (PERMANOVA) with the *adonis* function in Vegan package was used on Aitchison distance matrices generated by ALDEx2 package.^40–42^ ALDEx2 package was also used to calculate differential abundance of centered log-ratio transformed taxa counts, represented as effect size. Kruskal–Wallis test with post hoc Benjamini-Hochberg correction was used for statistical analysis.

*Functional abundance of SCFA-related enzymes*: The table of ASV counts and sequences of ASVs were input into PICRUSt2 for metagenomic prediction.^24,43-46^ Relative abundance of predicted sample gene family profiles was calculated using humann2_renorm_table function of HUMAnN2 pipeline.^47–49^ Statistical analysis was performed using Python package scipy and statsmodels. *P* values were adjusted using the Benjamini-Hochberg correction. A false discovery rate (FDR) of <0.05 was considered statistically significant.

### Behavioral assessment

Mice were acclimated in a conventional behavioral testing room for a minimum of 1 hour prior to initiation of any tests. Investigators were blinded to the treatment (aged or young microbiome) of each mouse and tests were conducted at the same time each day. Mice were allowed to recover 30 minutes between tests. The order of the tests for a given day was dependent on the perceived level of stress ranging from low to high stress. For instance, the tail suspension test was conducted last. To protect against cross contamination of bacteria between mice and to remove olfactory cues, all equipment was thoroughly cleaned with 70% ethanol between animals.

#### Novel objective recognition test

The novel object recognition test was performed at day 14 and 60 to investigate short-term cognitive function. Mice were placed in an arena with two identical objects for 10 min for a training period and then removed. After 1 h, mice were returned to the arena with a familiar object used in the training phase and a novel object. Exploration was assessed by monitoring the time spent with the novel object.

#### Open field test

The open field test was performed at day 14, 30 and 60. Mice were placed into separate arenas, each with a dimension of 16” x 16”. Locomotor activity of each mouse, and duration of activity was monitored for 20 min and total distance traveled and location in chamber was analyzed using Noldus EthoVision behavior software (Leesburg, Virginia).

#### Tail suspension test

The tail suspension test was performed by suspending the mice by their tail using a suspension apparatus. Animal activity and inactivity were recorded for the total duration of 6 min. The duration of inactivity during 6 min was a measure related to depressive-like behavior.

#### Hangwire test

The hangwire test was performed using a wired-cage top (18” x 9”). After the mouse was placed on the cage top, it was inverted 36 inches above soft bedding. Mice were monitored for a maximum duration of 5 min and the latency to fall from the inverted cage top was recorded as a measure of limb strength.

#### Barnes Maze test

Barnes Maze test was performed on an elevated circular platform with 20 evenly spaced holes. One of the holes was equipped with an escape box, allowing each mouse to escape and hide in the darkness. The maze was illuminated with bright overhead lights served as an aversive stimulus to encourage the mouse to escape. All training and testing for both groups were performed by the same blinded investigator. All behavior was recorded on video and analyzed using Noldus EthoVision behavior software (Leesburg, Virginia). Mice received 3 training trials for 2 minutes each. If a mouse failed to find the escape hole during the training trials, it was gently guided to the escape hole. The training trial was followed by a test trial (3 minute duration). The time taken to reach the entry zone, defined as a 3-cm border around the escape hole, was measured. The escape hole location was unchanged throughout the training and testing trials in order to allow for the mice to learn its location by the visual cues suspended around the maze.

### Metabolomics

Fecal and cecal samples were submitted to the Metabolomics Core at Baylor College of Medicine for processing and analysis. SCFAs were measured by LC-MS (Agilent LC-QQQ-MS system) and compared against a known reference. SCFAs were normalized to the measure obtained in the group with the young microbiome. When an SCFA was measured over several time points, the values were normalized to that obtained for the “30 day” time point for young microbiome.

### Brain cell analysis using flow cytometry

Ninety days after the initial FTG, mice were anesthetized with Avertin (250 mg/kg, i.p.) and blood was collected by cardiac puncture using a heparinized syringe. Each mouse was perfused transcardially using ice cold PBS solution and the brain was removed. The right hemisphere was homogenized and incubated in RPMI-1640 (Corning) containing 1 mg/ml of collagenase/dispase (Roche) and 10 mg/ml of DNase I (Roche) for 45 min at 37°C. The cell suspension was filtrated through a 70-μm cell strainer, washed and applied to a Percoll (GE Healthcare) gradients of 30% and 70% using the centrifugation at 500 g for 20 min. The interphase between the gradients was collected. Cells were stained with anti-CD45 (clone: 30-F11, BioLegend 103139), anti-CD4 (clone: RM4-5, BioLegend 100516), anti-CD8α (clone: 53–6.7, BioLegend 100737) and anti-TCRγδ (clone: GL3, BioLegend 118124). An amine reactive Live/Dead Aqua viability stain (Invitrogen L34966) was used to identify only live cells. Fluorescence-minus-one controls were used to distinguish positively stained cells for each antibody. The cells were analyzed using CytoFLEX (BECKMAN COULTER). The data were analyzed using FlowJo software (Tree Star, Inc.).

### Plasma cytokines

Cytokines were measured from plasma obtained 90 d after the initial FTG. Plasma samples were collected from both young and aged FTG groups at post-FTG day 90 (N = 10 for each group) using Bio-Plex Pro Mouse Cytokine Standard 23-Plex (Bio-Rad) according to the manufacturers protocol.

### Statistical analysis of behavioral testing and SCFAs

Two-way repeated measures ANOVA with post hoc Holm-Sidak test where appropriate (Figure 5, 6, 7 and Supplemental Figure IV) and the t-test (Supplemental Figure III, V, and VI) were used. *P* < .05 was considered statistically significant.Funding This work was supported by grants AG058436 from the National Institute of Aging (Bryan, McCullough, Co-PIs), NS103592 from the National Institute of Neurological Diseases and Stroke (McCullough, Bryan, Co-PIs), and grant HL134838 from the National Institute of Heart, Lung, and Blood (DJD), DK56338 from National Institute of Diabetes and Digestive and Kidney Diseases which supports the Texas Medical Center Digestive Diseases Center and its support to VRV and DJD; 15SDG23250025 from the American Heart Association (VRV); 19POST34410076 from the American Heart Association and the American Brain Foundation (JL). The metabolomics core was supported by CPRIT Proteomics and Metabolomics Core Facility (NP) (RP170005), NIH (P30-CA125123), and Dan L. Duncan Cancer Center.

## Supplementary Material

Supplemental Material
