# Supplementary material for: Young versus aged microbiota transplants to germ-free mice: increased short-chain fatty acids and improved cognitive performance
Source: Gut Microbes. 2020 Sep 8;12(1):1814107. doi: 10.1080/19490976.2020.1814107 (PMC7757789; doi:10.1080/19490976.2020.1814107)
Supplement: Supplemental Material [file KGMI_A_1814107_SM1679.pdf]

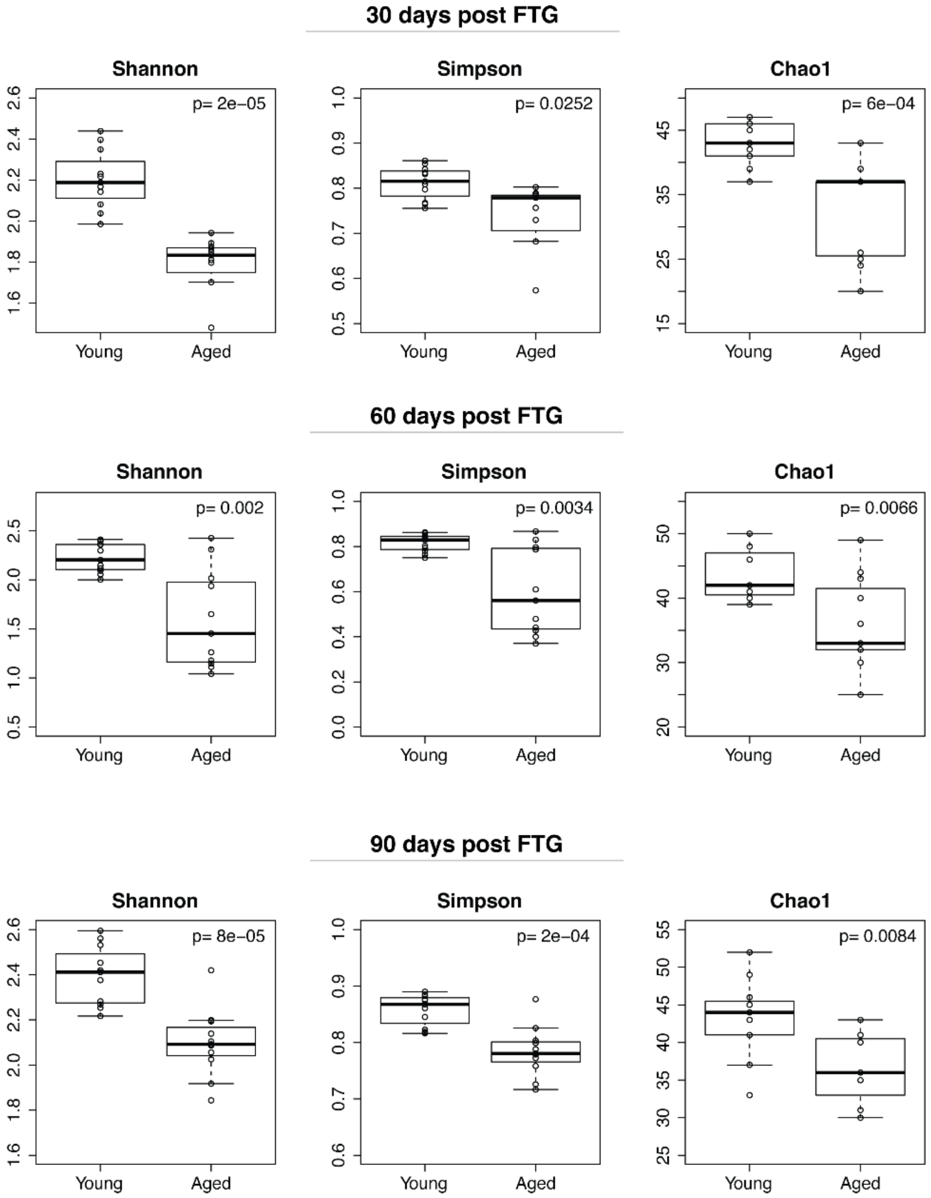

Supplemental Figure I. Alpha diversities (Shannon, Simpson, Chao1) at 30, 60, and 90 post-fecal transfer gavage (FTG). Data was analyzed using the t test with the adjusted P values in the upper right corner of each graph (N=11 per group). The Shannon and Simpson indices take into account both richness and evenness; Chao1 index only takes diversity into account.

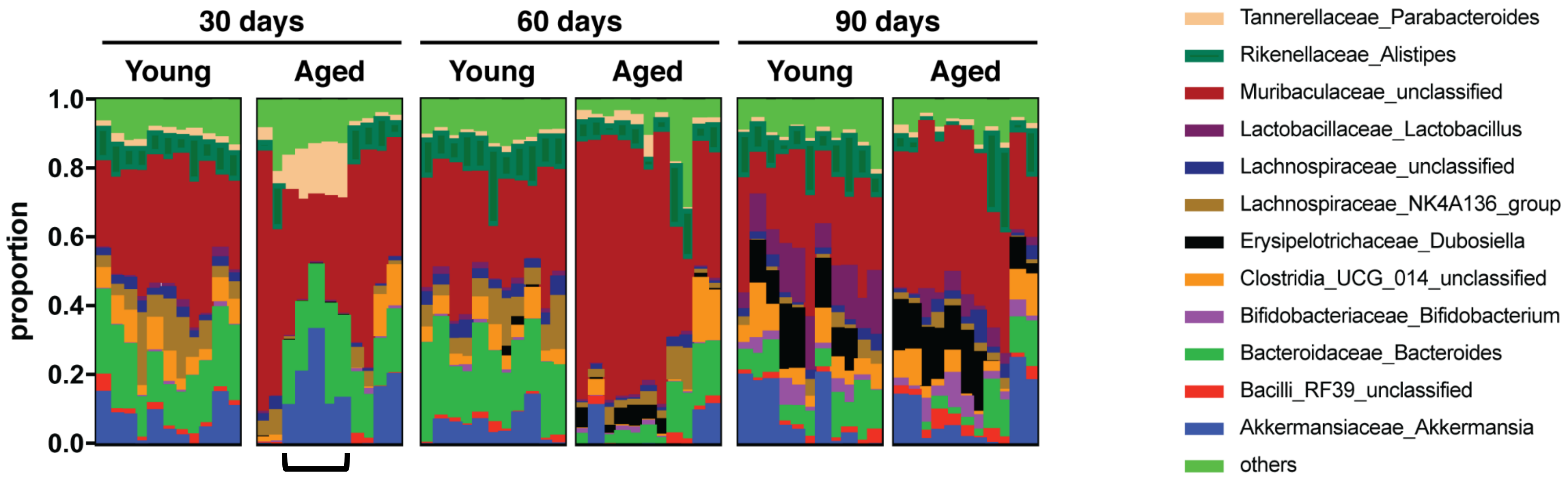

Supplemental Figure II: Stacked bar plots of taxa relative abundance for individual mice. Only taxa having >1% abundance in at least one group were included. The bracket below the aged group at 30 days represents a subset that separates from the others individuals in the same group (see text).

Main Effects of 2 way ANOVA				
**Figure**	Analysis for	Microbiome	Days after FTG	Microbiome x Days after FTG
**Figure 5**	Acetate	$F_{1,20}$ = 9.989, P=0.005	$F_{1,20}$ = 0.511, P=0.483	$F_{1,20}$ = 0.0975, P=0.758
**Figure 5**	Propionate	$F_{1,20}$ = 25.026, P<0.001	$F_{1,20}$ = 0.990, P=0.332	$F_{1,20}$ = 0.472, P=0.500
**Figure 5**	Butyrate	$F_{1,20}$ = 8.501, P = 0.009	$F_{1,20}$ = 5.173, P = 0.034	$F_{1,20}$ = 4.207, P = 0.054
**Figure 5**	Isobutyrate	$F_{1,20}$ = 3.695, P = 0.069	$F_{1,20}$ = 0.0214, P = 0.885	$F_{1,20}$ = 2.095, P = 0.163
**Figure 5**	2-methylbutyrate	$F_{1,20}$ = 3.526, P = 0.075	$F_{1,20}$ = 0.911, P = 0.351	$F_{1,20}$ = 1.839, P = 0.190
**Figure 5**	Valerate	$F_{1,20}$ = 3.641, P= 0.071	$F_{1,20}$ = 4.282, P = 0.052	$F_{1,20}$ = 14.482, P = 0.001
**Figure 5**	Isovalerate	$F_{1,20}$ = 0.644, P = 0.432	$F_{1,20}$ = 0.840, P = 0.370	$F_{1,20}$ = 1.031, P = 0.322
**Figure 6**	Novel Object Recognition	$F_{1,39}$ = 57.342, P < 0.001	$F_{1,39}$ = 1.531, P = 0.299	$F_{1,39}$ = 6.770, P = 0.003
**Figure 7A**	Tail suspension	$F_{1,60}$ = 8.804, P = 0.008	$F_{1,60}$ = 7.817, P <0.001	$F_{1,60}$ = 2.470, P = 0.071
**Figure 7B**	Barnes Maze	$F_{1,10}$ = 6.343, P = 0.030	$F_{1,10}$ = 7.252, P = 0.023	$F_{1,10}$ = 0.517, P = 0.489
**Supplemental Figure IVA**	Open field	$F_{1,40}$ = 1.394, P = 0.252	$F_{1,40}$ = 45.020, P <0.001	$F_{1,40}$ = 0.108, P = 0.898
**Supplemental Figure IVB**	Hang Wire	$F_{1,40}$ = 0.496, P = 0.489	$F_{1,60}$ = 7.112, P <0.001	$F_{1,60}$ = 1.057, P = 0.374

Supplemental Table I. Statistical table for all 2-way ANOVA in this study. F statistic and P value for all main effects [Microbiome, Days after FTG (fecal transfer gavage), and Microbiome X Days after FTG]. Subscript following F represents the degrees of freedom for the variables and the residuals respectively.

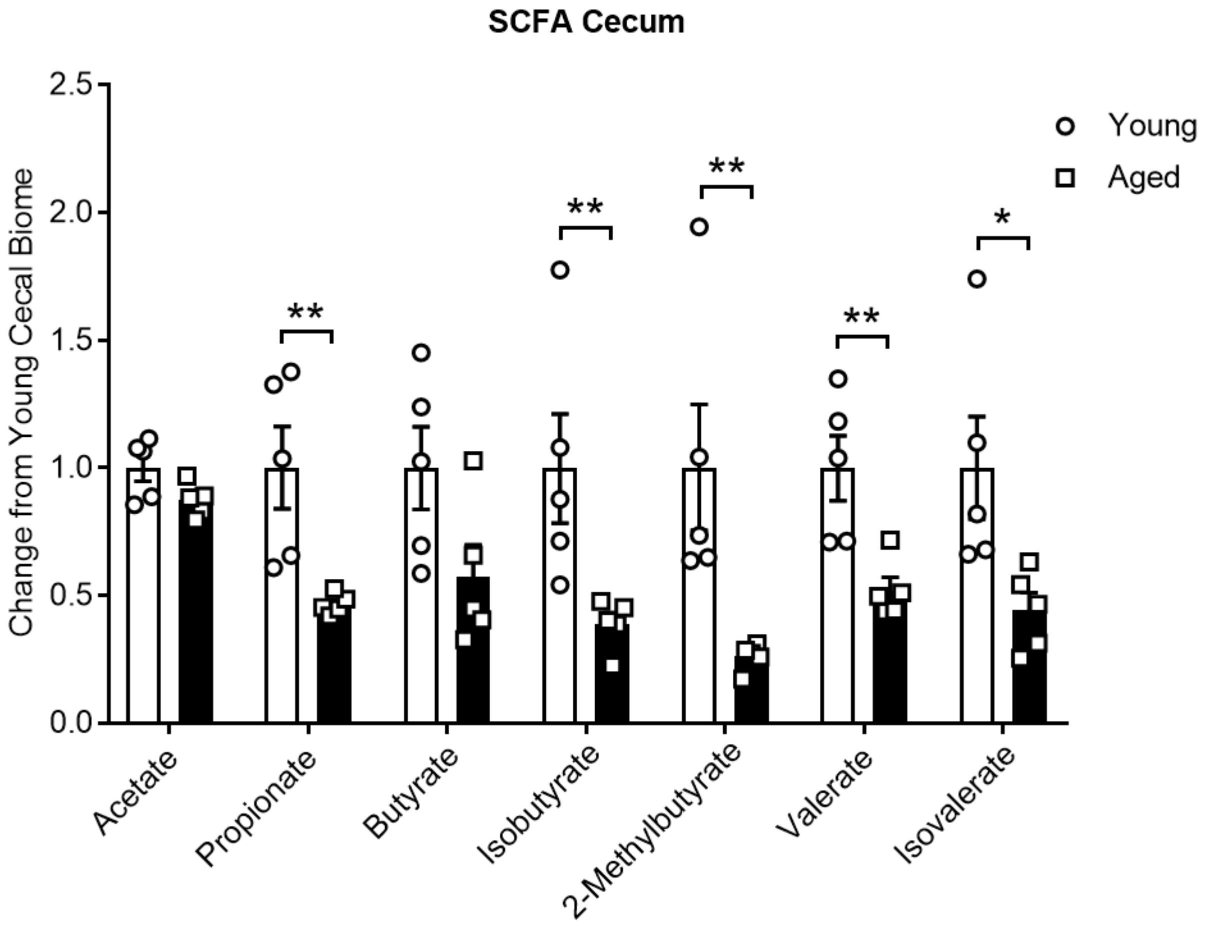

Supplemental Figure III. Changes in SCFA concentrations in cecal samples of mice with aged microbiomes relative to the concentrations in mice with young microbiomes at 90 days after initial FTG. *, ** P<0.05 and <0.01 respectively compared the young microbiome (t test, N=5 per group).

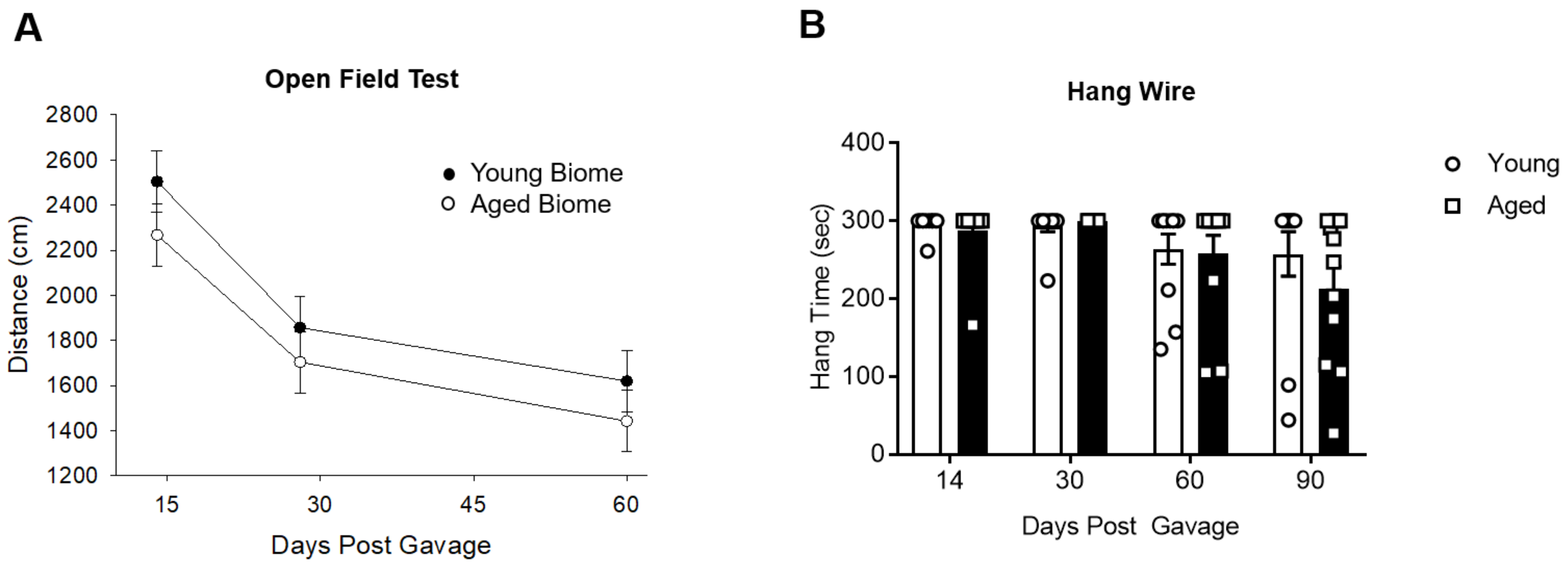

Supplemental Figure IV. (A) Open field test and (B) hang wire test. There were no significant differences between mice with a young or aged microbiome at any time point (N=11). In the Open field test there were no differences in either the total moving time or the velocity of movement between groups at any time point. See Supplemental Table I for more statistical detains.

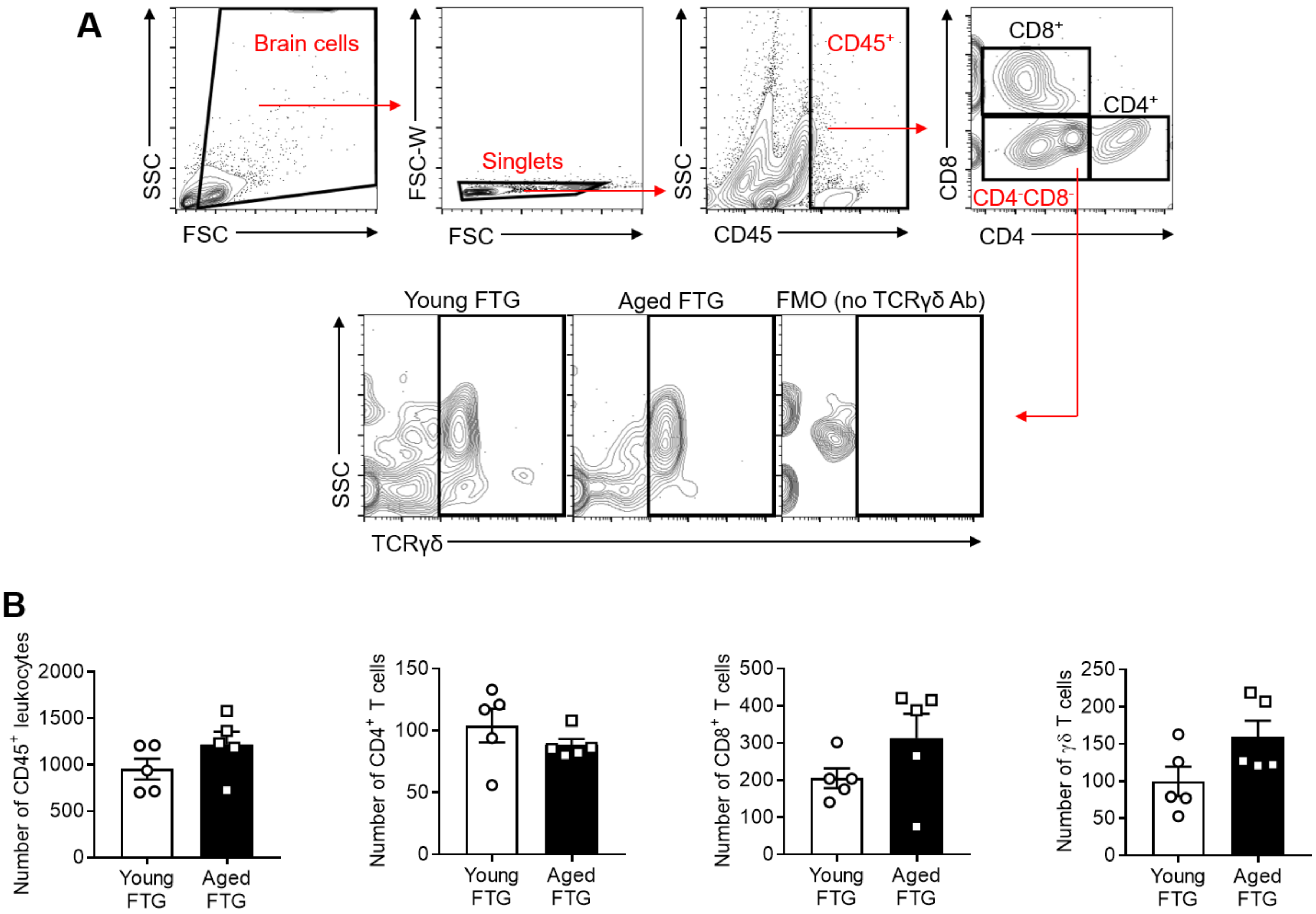
A
Brain cells
SSC
FSC
FSC-W
Singlets
CD45
CD45+
CD8
CD8+
CD4+
CD4-CD8-
CD4
Young FTG
Aged FTG
FMO (no TCRγδ Ab)
TCRγδ
B
Number of CD45+ leukocytes
2000
1500
1000
500
0
Young FTG
Aged FTG
Number of CD4+ T cells
150
100
50
Number of CD8+ T cells
500
400
300
200
Number of γδ T cells
250

Supplemental Figure V. (A) Gating strategy for flow cytometry studies from brains of mice that were removed and digested 90 days after the initial fecal transfer gavage. (B) There were no statistical differences in the number of $CD45^+$ cells (leukocyte), $CD4^+$ helper T cells, $CD8^+$ cytotoxic T cells, or γδ T cells ($CD45^+CD4^-CD8^-TCR\ \gamma\delta^+$) in brains from mice with a young and aged microbiome (n=5 per group).

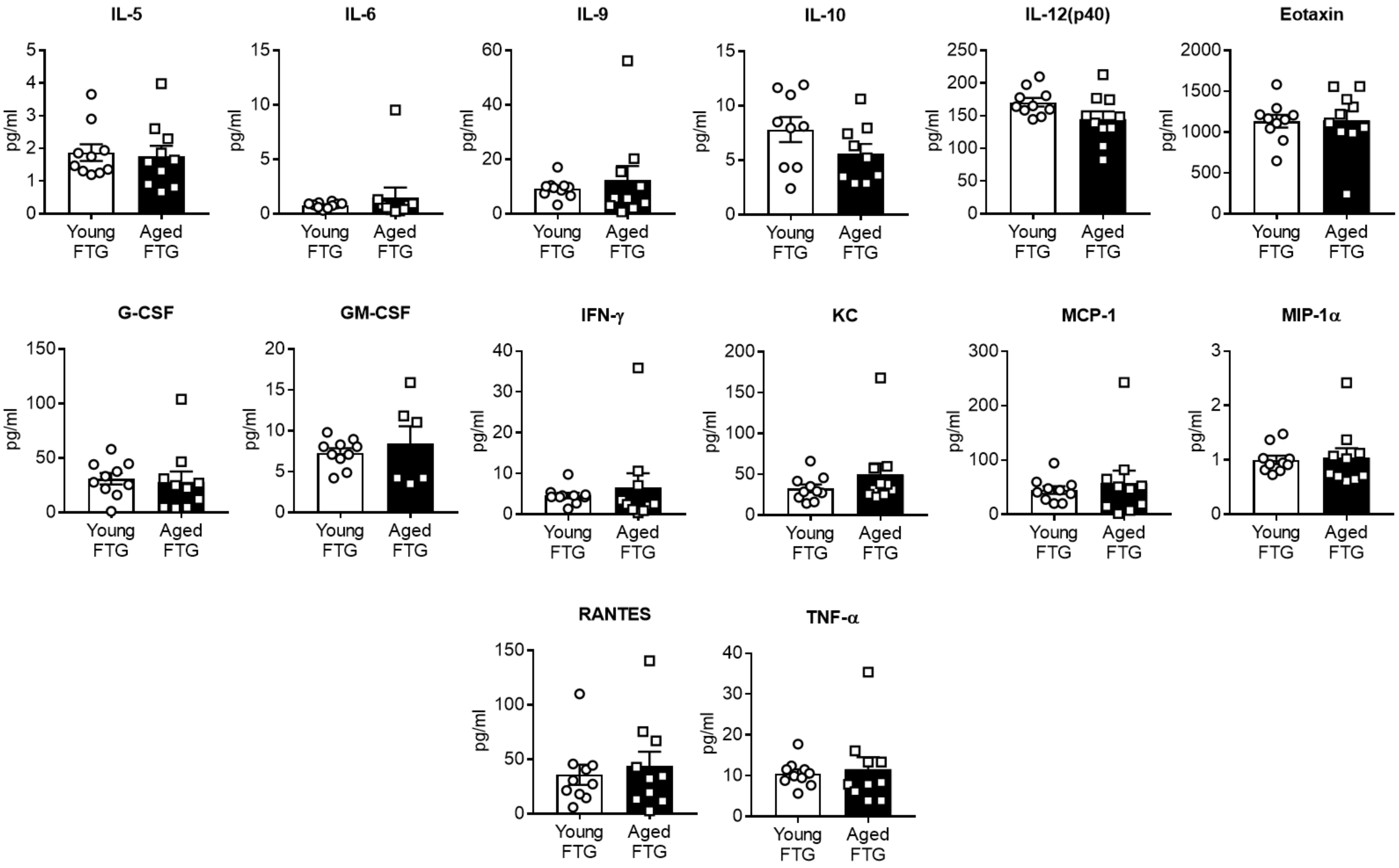
IL-5
IL-6
IL-9
IL-10
IL-12(p40)
Eotaxin
G-CSF
GM-CSF
IFN-γ
KC
MCP-1
MIP-1α
RANTES
TNF-α
pg/ml
Young FTG
Aged FTG

Supplemental Figure VI. Plasma cytokine profiles at 90 days after the initial fecal transfer gavage in mice with a young or aged microbiome (N=6-10 per group). There were no significant differences in IL-5, IL-6, IL-9, IL-10, IL-12(p40), eotaxin, granulocyte colony-stimulating factor (G-CSF), granulocyte-macrophage colony-stimulating factor (GM-CSF), IFN-γ, keratinocyte chemoattractant (KC, also known as CXCL1) or, monocyte chemoattractant protein-1 (MCP-1), macrophage inflammatory protein-1α (MIP-1α), RANTES or TNF-α.
